# Fasciotomy on a female patient with acquired factor VIII: A case report and literature review of management

**DOI:** 10.1002/ccr3.7773

**Published:** 2023-08-03

**Authors:** Katie Lovell, Bethlehem Peters, Melisa Pasli, Katie Kennedy, Darla Liles, Walter Pories

**Affiliations:** ^1^ East Carolina University Brody School of Medicine Greenville North Carolina USA; ^2^ Department of Hematology/Oncology ECU Health Brody School of Medicine Greenville North Carolina USA; ^3^ Department of Surgery ECU Health Brody School of Medicine Greenville North Carolina USA

**Keywords:** acquired hemophilia, compartment syndrome, factor VIII inhibitors, fasciotomy, hematology, immunology, surgery

## Abstract

**Key Clinical Message:**

Acquired factor VIII inhibitors can be a rare cause of extensive intramuscular bleeding requiring fasciotomy. The subsequent postoperative period requires close monitoring due to high risk of fatal blood loss.

**Abstract:**

Acquired factor VIII inhibitors are a rare cause of often extensive bleeding and subsequently large hematomas. This disorder's overall mortality can reach 38%, largely due to immunosuppression and subsequent infections or an underlying cause such as malignancy. The patient in this case study presented with a hematoma and extensive ecchymosis of the hand and forearm, which continued to progress, precipitating compartment syndrome of the hand and forearm and ultimately requiring fasciotomy. The combination of factors led to significant blood loss in the postoperative period requiring major fluid resuscitation and intensive care unit (ICU) level care. Due to this disorder's rarity and overall mortality, we present this case report with a literature review for management of acquired hemophilia in the setting of urgent fasciotomy.

## Graphical abstract

Trending the hemoglobin levels over the hospital course of a patient presenting with Acquired Hemophilia A who developed compartment syndrome secondary to intramuscular bleeding with subsequent emergent fasciotomy performed.

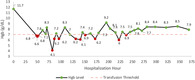

## 1 INTRODUCTION

Acquired hemophilia A (AHA) is a rare bleeding disorder characterized by the development of autoantibodies to factor VIII, causing large cutaneous hematomas and severe muscle bleeding. It often presents in patients with no personal or familial history of bleeding.^1^ AHA risk factors are associated with autoimmune diseases, the postpartum period, malignancy, and increased age; though 50%–60% of cases present without an identified etiology.^2^ Certain medications are also associated with the development of AHA, such as DPP‐4 inhibitors or immune checkpoint inhibitors.^1^ The estimated incidence of this disorder is 1–4 patients per million, though the incidence is increasing due to increased recognition and recently published recommendations on diagnosis and management.^1^ Recently, there has been a documented association between primary AHA and reemergence after SARS‐CoV‐2 infection.^3^, ^4^, ^5^ This acquired bleeding disorder can be difficult to diagnose and treat. Thus, transfer to tertiary institutions is often required to manage the potentially fatal bleeding with appropriate agents and lab monitoring.^2^

Compartment syndrome can be a sequelae of AHA and is a surgical emergency. However, the literature is unclear on when fasciotomy is safest in atraumatic acute compartment syndrome secondary to AHA. Additionally, atraumatic compartment syndrome of the hand secondary to AHA is exceedingly rare, and to our knowledge, one prior case report has been published.^6^ We present a case of atraumatic acute compartment syndrome secondary to AHA in a 70‐year‐old female, requiring emergent hand and forearm fasciotomy with complete motor function recovery. The patient course included significant hemorrhage and transfusion of blood products.

## 2 CASE REPORT

A 70‐year‐old female was transferred to our hospital for concern of right upper extremity (RUE) compartment syndrome secondary to an expanding hematoma. Medical history was significant for type 2 diabetes, coronary artery disease, congestive heart failure, history of transient ischemic attack (TIA), sleep apnea, and anemia of chronic disease. She was taking ticagrelor due to her TIA and cardiac history but had no prior history of bleeding. The patient presented to the outside hospital 2 days before admission to our institution due to right‐hand pain, itching, and swelling for 2 days without trauma to the area. Erythema of the right hand was noted, and the patient was started on intravenous (IV) rocephin 2 g due to concern for cellulitis. During her initial admission, edema of the right hand and arm increased, and a hematoma developed in the antecubital region after an IV stick in the region. The patient was anemic with a hemoglobin (Hgb) of 6.8 g/dL prior to transfer, losing 5 g/dL of blood from 11.7 g/dL at presentation. Coagulation studies for prothrombin time (PT) and international normalized ratio were within the normal range. Partial thromboplastin time (PTT) was prolonged, but mixing studies were not able to be completed (Table 1). The patient could not receive cryoprecipitate, fresh frozen plasma (FFP), or platelets prior to transfer. Additionally, she had increased pain, swelling, ecchymoses, and a decreased palpable pulse of the RUE, prompting the transfer for further evaluation and treatment of compartment syndrome. Upon arrival, the patient was anemic at 6.8 g/dL, and coagulation studies revealed a severely elevated activated PTT (aPTT) at 92.1 s. The patient had significantly increased swelling, pain, and intermittent paresthesia on physical exam, prompting urgent fasciotomy.

**TABLE 1:** Admission, post‐anesthesia care unit (PACU), postoperative day 1 (POD#1), and discharge laboratory values (top) and specialized tests for acquired hemophilia initially at discharge, and at 1 month follow‐up (bottom).

Test	Admission values	PACU levels	POD#1	Discharge values	Reference range
Hemoglobin (Hgb)	6.8	8.3	4.1	7.9	13.0–18.0 g/dL
Hematocrit (Hct)	20.7	24.5	12.3	24	35%–47%
Red blood cell distribution width (RDW)	15.4	14.6	14.1	15.4	11.5%–14.5%
Platelet count (Plt)	247	288	190	344	150–440 k/μL
Prothrombin time (PT)	11.8		12.4		10.2–12.9 s
Activated partial thromboplastin time (aPTT)	92.1		57.8		25.1–36.5 s

Test	Initial values	Discharge values	1 month follow‐up	Reference range
Factor VIII%	1	52	310	50%–150%
Bethesda inhibitor	4	2	0	0 BU

In the operating room (OR), compartment pressures were measured with the Stryker (STIC) device, which showed elevated pressures to the 50–60 s mmHg in the volar forearm compartment, dorsal forearm, and dorsal hand. Thenar and hypothenar pressures were also elevated above 30 mmHg. The delta pressure above the elbow was evaluated to be within normal limits. Complete fasciotomies of the volar and dorsal hand and forearm were completed. Blood loss during the operation was approximately 500 cc, and the wounds were left open due to swelling preventing even partial closure of the area. The patient received 2 units of packed red blood cells (pRBC) in the OR, followed by 1 unit of pRBCs and 250 cc of albumin in the post‐anesthesia care unit.

In the postoperative period, the arm dressing became saturated with serosanguinous fluid. The patient then received 2 units of platelets, 2 FFPs, and 1 unit of pRBCs. An internal jugular central line was placed without complications, and the patient was transferred to surgical intensive care unit (SICU). Upon arrival at SICU, the patient was hypotensive, and resuscitation with FFP, platelets, and cryoprecipitate was initiated. Her repeat thromboelastographic (TEG) test with R time was down to 22 s, and an additional units of FFP and 1 unit of platelets were ordered. The patient's dressings were taken down, and muscles were determined to be viable‐ oozing from the skin edges were noted. The wound was repacked with QuikClot. The patient remained hypotensive in the early postoperative period and required pressor support of levophed (16 mcg/mL) for 8 h, which titrated from a range of 7.5 mL/h to 30 mL/h to maintain mean arterial pressure of 65 mmHg. On postoperative day (POD) #1, the patient's hemoglobin dropped from 7.8 to 4 g/dL; she was given an additional 2 units of blood at that time. Due to hemodynamic instability and swelling of the area, return to the OR for closure/partial closure was delayed until POD#5.

**FIGURE 1:**
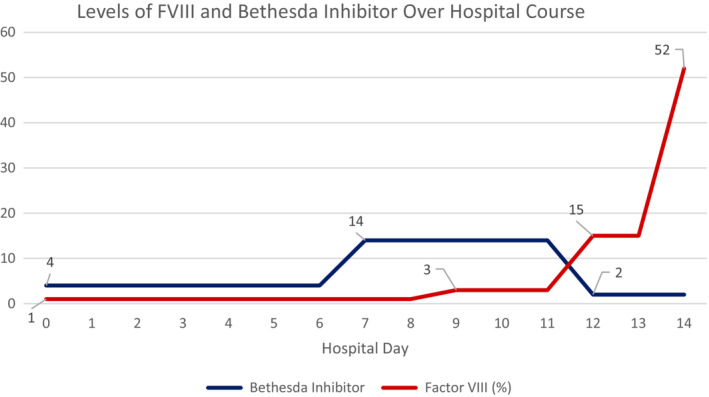
Bethesda inhibitor level versus factor VIII level over the hospital course.

In addition to the close lab monitoring of hemoglobin/hematocrit, coagulation, TEG, and mixing studies were ordered in the postoperative period. Mixing studies showed severely low factor VIII levels (1%), and hematology/oncology was consulted at that time. Due to the patient presentation and suspicion for AHA, prothrombin complex concentrate (PCC) was initiated at 50 U/kg and a Bethesda inhibitor titer was ordered and was subsequently positive at 4 Bethesda units (BUs) (Figure 1). PCC immediately slowed the bleeding, and the dose was increased to 75 U/kg over 6 or 8 h. Due to the presence of the factor VIII inhibitor, rituximab 375 mg/m^2^ × 4 weekly doses, cyclophosphamide 50 mg, and prednisone 1 mg/kg were started to assist in the elimination. Despite receiving PCC earlier on POD#1, the patient had persistent oozing of the surgical site, and Hgb decreased from 7.2 to 6.2 g/dL. She received 2 units of pRBC and Lasix was given between units. She remained hemodynamically stable without vasopressors. Hemoglobin responded appropriately and increased to 8.3 g/dL on POD#2. PCC dosing was weaned to 75 U/kg over 12 h; however, bleeding recurred after the reduction in frequency and subsequent dressing removal. At that time, 1 unit of an anti‐inhibitor coagulant complex (FEIBA) was ordered 50 U/kg every 8 h in place of PCC, but subsequent units of FEIBA could not be administered due to a lack of supply over the weekend. Hemoglobin dropped again to 6.1 g/dL on POD#3, and the patient received 2 units of pRBC at separate times. On POD#4, FEIBA administration was restarted and increased to 100 U/kg q12 h on POD#5.

The patient returned to the OR for fasciotomy closure on POD#5. During the procedure, large clot formations were removed from the volar aspect of the forearm muscles. Persistent ooze from suturing needle sites, skin edges, and muscle bellies was noted with two to three areas of small vessel bleeding that were coagulated. There was a total of 350 cc blood and blood clot loss during the procedure. After clot evacuation, the area was irrigated with warmed saline and 0.5 L of dilute chlorhexidine solution (Irrcept) before a final wash with warmed saline. The entire wound area was treated with a thrombin spray before serial closure with a combination of buried 3–0 PDS interrupted dermal sutures and interrupted 3–0 Prolene skin sutures. Oozing of the suture sites and incision persisted after closure, and the area was covered with thrombin spray soaked QuikClot gauze along with 4 × 4 s, abdominal gauze pads, and wrapped with Kerlix and ACE wrap. She received an additional pRBC and cryoprecipitate in the OR. Postoperatively, the patient received a dose of PCC in addition to her evening dose of FEIBA.

**FIGURE 2:**
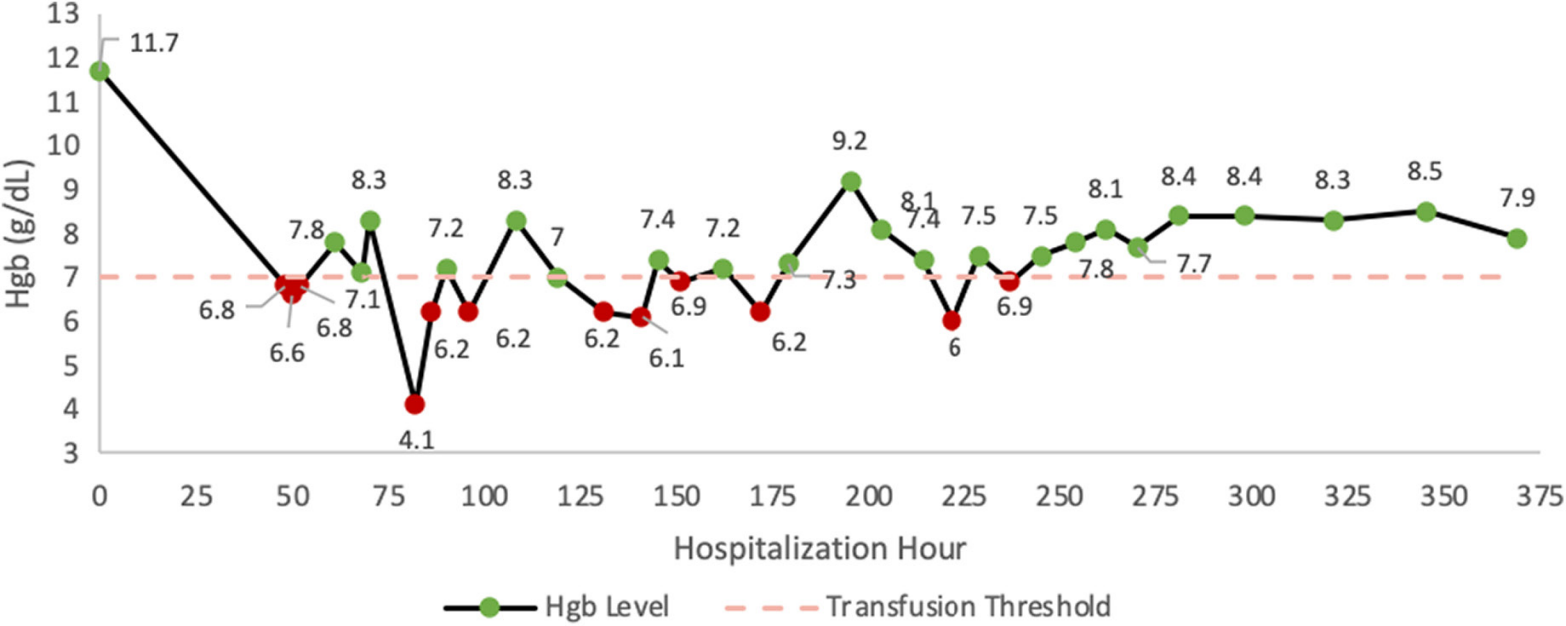
Hemoglobin levels over the hospital course with green points representing levels >7.0 g/dL and red points representing levels <7.0 g/dL, which is below the transfusion threshold (denoted by the orange dashed line at g/dL = 7.0).

After closure of the fasciotomy, hemoglobin started to stabilize—measured at 8.1 g/dL 1 day after closure. Oozing from the suture sites was still present, but overall bleeding had significantly decreased, and the patient continued to receive FEIBA 100 U/kg every 12 h. There was a final drop in Hgb on POD#2, requiring 2 units of pRBCs, and wound dressings were changed due to saturation (Figure 2). FEIBA was discontinued on POD#5 from closure due to stabilized Hgb and lack of bleeding from the incision. Rituximab 375 mg/m^2^ × 4 weekly doses, cyclophosphamide 50 mg daily, and prednisone 1 mg/kg daily were continued. On POD#6, the factor VIII level was increased to 15%, and 2 days later, the level was increased to 52%. Our patient required 13 units of pRBCs during her stay. The patient was discharged from our institution with a follow‐up in 1 week for her final rituximab transfusion. She was discharged on cyclophosphamide and prednisone until her follow‐up transfusion appointment.

Five days after discharge, the patient presented to an outside hospital's emergency department after noticing light saturation on her surgical dressing. She was evaluated to have mild serosanguinous fluid from the proximal dorsal forearm likely secondary to increased arm usage. Lab values were significant for slightly elevated aPTT at 33.5 s, decreased Hgb at 10.1 g/dL, and decreased hematocrit at 30.5%. Her dressings were replaced, and she was discharged with Keflex due to the presence of some mucus discharge around the wound. Despite some minor complaints of nerve pain, sensory and motor function was evaluated to be normal. At 1 week after discharge, she received the final rituximab infusion, and at her 1‐month follow‐up with hematology/oncology, her lab values revealed an elevated factor VIII level (310%) and a negative Bethesda inhibitor assay. Due to these findings, cyclophosphamide and prednisone were discontinued with follow‐up in 3 months to reassess labs.

## 3 DISCUSSION

With a mortality rate as high as 33%, early detection and treatment of AHA are essential in decreasing patient morbidity and mortality.^6^ The rarity of AHA is a barrier to standardizing care. In recent years, the increase in case reports and diagnostic management guidelines has allowed for early identification and management improvements. However, a paucity of cases limits the widespread knowledge of the standard practice. AHA should be considered in acute atraumatic compartment syndrome cases, particularly in those with risk factors such as advanced age, recent COVID‐19 infection, and DPP‐4 inhibitor use. Early recognition of AHA in acute compartment syndrome is imperative due to the high risk of postoperative bleeding. In this case, AHA was not diagnosed until after the fasciotomy due to an investigation of ongoing hemorrhage.

In this case, risk factors for developing AHA were advanced age, use of DPP‐4 inhibitors, and COVID‐19 infection in the past 3 months. A previous case series demonstrated that utilization of DPP‐4 inhibitors for treatment of diabetes mellitus has the potential to induce AHA^7^ due to the generation of autoantibodies to factor VIII. While our patient did not have a history of malignancy or autoimmune disease, there have been numerous case reports discussing the association between COVID‐19 infection and subsequent development of AHA.^3^, ^4^, ^5^, ^8^ She received three doses of the Moderna COVID‐19 vaccine prior to her infection and received an additional booster 1 month after her recovery (1.5 months prior onset of AHA).

Typically, patients with AHA present with acute bleeding patterns and no prior bleeding history. In the order of incidence, patterns of bleeding typically seen in AHA are subcutaneous, muscle, gastrointestinal, genitourinary, and retroperitoneal^9^; contrarily, hemarthroses are characteristics of congenital Hemophilia A and not associated with AHA.^9^ Diagnosis of AHA requires PTT and PT values. If only PTT is prolonged, a mixing study is done with 1:1 patient plasma: normal plasma; persistent PTT prolongation after the mixing study requires quantification of coagulation factors to look for an inhibitor. Decreased levels of FVIII are suggestive of AHA. Subsequent antibody titers should be obtained utilizing the Nijmejen modification of the Bethesda assay.^1^

Treatment of AHA bleeding requires activated PCC (aPCC, FEIBA) alone or with recombinant FVIIa, which work by bypassing FVIII in the coagulation pathway.^1^ aPCC can be dosed multiple times per day at 50–100 U/kg of body weight with a maximum of 200 U/kg per day and should be continued until clinical signs of bleeding are no longer evident. If aPCC or FVIIa fail to stop the bleeding, an alternate agent is recommended, and in the case of persistent bleeding, recombinant porcine FVIII can be used. Corticosteroids (prednisone 1 mg/kg for 4–6 weeks) are the primary management in eliminating the autoantibody. Cyclophosphamide can be added, especially in the setting of significantly deficient FVIII levels (<1%) and high antibody titer (>20 BU).^1^, ^9^ Rituximab (375 mg/m^2^ once weekly for 4 weeks) can be used in addition to these agents.^1^ A combination of agents, such as prednisone and cyclophosphamide or prednisone and rituximab, may decrease the time to remission.^1^ Efforts to treat the underlying cause should also be considered in cases with a known etiology to reduce the risk of recurrence.^1^ While platelet transfusions are not standard in AHA management, the patient in this study received platelets during early resuscitation efforts secondary to a rapid, acute drop in hemoglobin without knowledge of the patient's final diagnosis.

Fasciotomy in the setting of AHA is a limb‐saving but risky procedure, which emphasizes the importance of early diagnosis of AHA. The primary risk of fasciotomy in a patient with AHA is excessive perioperative bleeding that is difficult to manage due to the presence of a clotting factor inhibitor. In a review of current literature, the cases with the shortest length of stay and use of blood products were cases with early diagnosis and intervention for AHA.^10^, ^11^, ^12^ The case reports from Bruggers and Adeclat demonstrated the utility of early initiation of AHA treatment to attempt to avoid or delay fasciotomy.^10^, ^11^ While the patients in these reports ultimately required fasciotomy, the length of stay was limited with a decreased report of required blood products.^10^, ^11^ Likewise, cases where AHA diagnosis was not determined until the postoperative period resulted in increased number of blood products, urgent exploratory surgeries, and increased length of hospital stays.^6^, ^13^, ^14^ The patient in this study likely falls between the two extremes as discussed. While her diagnosis did not occur until the postoperative period, early consultation with hematology/oncology helped stabilize her condition and initiate appropriate treatment.

## 4 CONCLUSION

The goal of this report and subsequent literature review is to add to the knowledge base of AHA management in the surgical setting. Collaboration between surgical and medical physicians can help promote early detection of AHA and improve patient outcomes. The presence of large, atraumatic hematomas in a patient with associated risk factors and without prior bleeding history should prompt evaluation for coagulation pathway abnormalities. As demonstrated by this review, aggressive resuscitation, repletion of low clotting factors, and elimination of the autoantibody decrease length of stay and need for higher level care. While fasciotomy is often a necessary treatment in this setting, care should be taken to thoroughly workup a patient for clotting factor abnormalities with appropriate fluid replacement prior to surgery.

## AUTHOR CONTRIBUTIONS

**Katie Lovell:** Conceptualization; formal analysis; project administration; visualization; writing – original draft; writing – review and editing. **Bethlehem Peters:** Conceptualization; formal analysis; writing – original draft; writing – review and editing. **Melisa Pasli:** Conceptualization; writing – original draft; writing – review and editing. **Katie Kennedy:** Supervision; writing – review and editing. **Darla Liles:** Supervision; validation; writing – review and editing. **Walter Pories:** Supervision; validation; writing – review and editing.

## FUNDING INFORMATION

This research received no specific grant from any funding agency in the public, commercial, or not‐for‐profit sectors.

## CONFLICT OF INTEREST STATEMENT

No external funding or competing interests are declared. The authors listed have no conflicts of interest to disclose.

## CONSENT

This report was completed with the written consent of the patient.

## Data Availability

Data sharing is not applicable to this article as no new data were created or analyzed in this study.
